# The prevalence of Internet addiction and its relationship with sociodemographic characteristics

**DOI:** 10.1192/j.eurpsy.2025.987

**Published:** 2025-08-26

**Authors:** M. Rmili, S. Boulbaroud, M. El Ayachi, A. Chakir Lamrani, H. Hami, F.-Z. Azzaoui

**Affiliations:** 1 Laboratory of Biology and Health, Faculty of Sciences, Ibn Tofail University, Kenitra, Morocco; 2 Unit of Biotechnology and Sustainable Development of Natural Resources, Polydisciplinary Faculty, Sultan My. Slimane University, Beni Mellal, Morocco

## Abstract

**Introduction:**

The increasing integration of the Internet into daily life has raised numerous concerns, including the risk of addiction. This addiction may be influenced by sociodemographic factors.

**Objectives:**

Determinate the prevalence of Internet addiction among adolescents and examine the effect of sociodemographic factors on Internet addiction.

**Methods:**

This is a cross-sectional study with a descriptive and analytical aim in the Oued eddahab high school in the city of Tiflet of the Khemisset provincial direction, including 378 students (239 girls and 139 boys), the average age of the participants is 17.0.8±1.28 years. The Students were asked to complete a socio-demographic questionnaire and an internet addiction test (IAT) consisting of 20 items to determine the level of addiction.

**Results:**

The prevalence of Internet addiction among students is 55.6%. The addiction of these students is highly significantly related (χ2=22.893, p<0.001) to conflicts with their mothers. While conflicts with their fathers are highly significantly related (χ2=12.961, p<0.01) to Internet addiction. However, conflicts between parents have a highly significant relationship (χ2=9.421, p<0.01) with Internet addiction. Parents’ control of Internet access has a highly significant influence (χ2=10.473, p<0.01) on Internet addiction.

**Conclusions:**

Students who experience conflicts with their mothers, fathers, or between their parents are more likely to develop Internet addiction. Parental control over Internet access helps reduce the risk of addiction.

**Disclosure of Interest:**

None Declared

